# Control and Prevention of Viral Gastroenteritis

**DOI:** 10.3201/eid1708.110824

**Published:** 2011-08

**Authors:** Stephan S. Monroe

**Affiliations:** Author affiliation: Centers for Disease Control and Prevention, Atlanta, Georgia, USA

**Keywords:** norovirus, rotavirus, viruses, foodborne illness, viral gastroenteritis, enteric infections, prevention, control, perspective

## Abstract

Diarrheal illness remains 1 of the top 5 causes of death in low-income and middle-income countries, especially for children <5 years of age. Introduction of universal childhood vaccination against rotaviruses has greatly reduced the incidence and severity of illness in upper-income and lower-income settings. For adults, norovirus is the leading cause of sporadic cases and outbreaks of diarrheal illness and is responsible for nearly 21 million episodes annually in the United States, of which 5.5 million are foodborne. Public health efforts to control and prevent norovirus illness have focused on rapid outbreak detection and source identification and control of transmission in institutional settings.

Diarrheal illness remains 1 of the top 5 causes of death in lower-income and middle-income countries (*1*), especially for children <5 years of age. In the ≈40 years since the initial detection of Norwalk virus (*2*) and rotavirus (*3*) by electron microscopy in stool samples of patients with gastroenteritis, there has been increased recognition of the role of enteric viruses as a major cause of diarrhea-associated illness and death in young children and adults. Unfortunately, standard improvements in water and sanitation that reduce the incidence of enterically transmitted bacteria do not appear to be equally effective for reducing the incidence of enterically transmitted viruses. Thus, other public health approaches have been pursued for the control and prevention of viral gastroenteritis.

For children <5 years of age, rotavirus is the leading cause of diarrhea-associated illness and death. Fortunately, safe and effective vaccines against rotavirus illness are now available in many countries. Introduction of universal childhood vaccination against rotaviruses greatly reduces the incidence and severity of illness in upper-and lower-income settings (*4*). As a result, the World Health Organization recommended in 2009 that rotavirus vaccines be included in all national immunization programs (*5*).

In adults, norovirus is now recognized as the leading cause of sporadic cases (*6*) and outbreaks of diarrheal illness and is responsible for ≈21 million episodes annually in the United States, of which 5.5 million are foodborne (*7*). Efforts to develop effective vaccines for norovirus have been hindered by lack of a cell culture system to propagate the virus, large genetic diversity of norovirus strains, and apparent lack of long-term immunity generated by natural infection. Recent work on characterizing the interaction between noroviruses and their putative cellular receptors, histo–blood group antigens, may provide insights for development of specific antiviral compounds (*8*).

Public health efforts to control and prevent norovirus illness have focused primarily on outbreak detection and control. The implementation of CaliciNet, as described by Vega et al. (*9*), provides a useful new public health tool for rapid identification of norovirus outbreaks. Similar to the successful PulseNet network for molecular typing of foodborne bacteria (*10*) and NoroNet in Europe (*11*), CaliciNet will enable linking of cases with identical sequence fingerprints into clusters of illness that may have a common exposure. This linking will be particularly useful in cases of illness related to food products with low levels of contamination in which identification of exposure to a common food source may be difficult by epidemiologic methods alone.

Because a large proportion of norovirus illness results from foodborne exposures, considerable effort has gone into development of methods for detecting and eliminating virus contamination from food items, particularly shellfish (*12*) and fresh produce (*13*). Additionally, because outbreaks of norovirus illness often occur in institutional settings, efforts are under way to standardize effective methods for disinfection of contaminated surfaces (*14*).

Finally, several other viruses, including astrovirus, sapovirus, and as described by Drexler et al. (*15*), Aichi virus, are also responsible for diarrheal illness in children and adults. Although the incidence and severity of illness caused by these pathogens may not warrant immediate development of vaccines, work continues to document their relative contributions to diarrhea-associated illness and death. Thus, although there is optimism for universal vaccination to prevent illness and death from severe rotavirus diarrhea and for reduction of norovirus illness by rapid outbreak detection and source identification, there are still many challenges remaining for the control and prevention of viral gastroenteritis.
